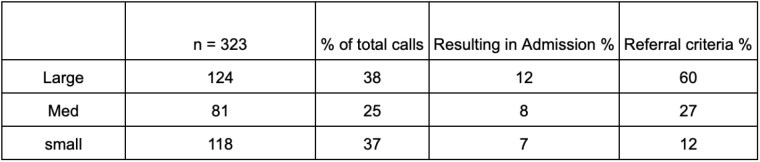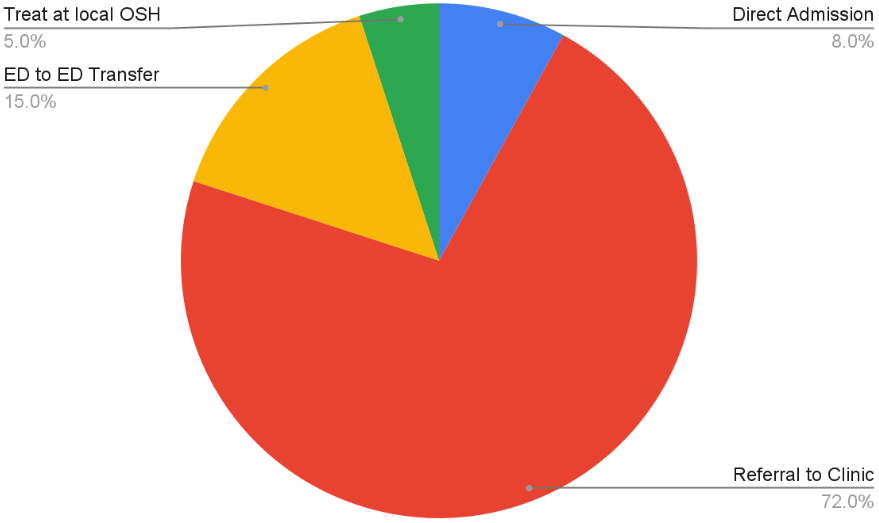# 660 Calling the Burn Center: Why Do Our Referring Hospitals Call Us?

**DOI:** 10.1093/jbcr/iraf019.289

**Published:** 2025-04-01

**Authors:** Moon Usman, Alisa Savetamal

**Affiliations:** Connecticut Burn Center, Bridgeport Hospital / Yale-New Haven Health; Connecticut Burn Center, Bridgeport Hospital / Yale-New Haven Health

## Abstract

**Introduction:**

This study seeks to analyze and evaluate consultation and referral data received by a single state burn center, focusing on the allocation of resources for referrals and consultations, and determining the proportion of these that result in patient admissions.

**Methods:**

We conducted a retrospective review of call data to the burn center to examine the frequency and nature of calls (consultation versus referral for admission) and compare estimated Total Body Surface Area (TBSA) with actual calculated TBSA%. Calls were categorized based on patient disposition decisions (e.g., direct transfer, referral to clinic, referral to our ED for further evaluation, follow-up with primary care physician). We analyzed the total call data, admission rates, and referral criteria.

**Results:**

During an eight-month period, our burn center received 323 telephone inquiries regarding burn-injured patients at other facilities. We observed that the proportion of calls from larger hospitals (those with over 300 beds) was similar to that of small hospitals, at 38% and 37% respectively. However, larger institutions were more likely to meet the referral criteria compared to smaller hospitals, which, while calling at a similar frequency, were less likely to lead to admissions. Overall, only 8% of all referrals resulted in direct admissions (see Table 1). In terms of staff resources, we noted an average of 40 calls per month, with evaluation times varying from 10 to 30 minutes, resulting in approximately 13 hours per month dedicated to this activity.

**Conclusions:**

Communicating with referring institutions about burn care is a daily part of running a burn center. In our experience, however, a significant number of these calls will not directly result in patient admissions. It is important to quantify the time consumed in this work, which often is effectively an uncompensated service, so that proper staffing can be calculated. Second, this review reinforces the need for ongoing education for healthcare professionals and the general public. Ongoing education and outreach are imperative not only to optimize the utilization of burn centers but also to foster a collaborative approach among healthcare providers. The synergy between specialized burn centers and broader healthcare education is essential for advancing the quality of care in this critical area.

**Applicability of Research to Practice:**

This research highlights the importance of burn centers as a resource for referring hospitals, as well as the need for continued outreach and education.

**Funding for the Study:**

N/A